# Supplementary material for: Modelling the impact of the tier system on SARS-CoV-2 transmission in the UK between the first and second national lockdowns
Source: BMJ Open. 2021 Apr 22;11(4):e050346. doi: 10.1136/bmjopen-2021-050346 (PMC8068949; doi:10.1136/bmjopen-2021-050346)
Supplement: Author's manuscript [file bmjopen-2021-050346.draft_revisions.pdf]

BMJ Open is committed to open peer review. As part of this commitment we make the peer review history of every article we publish publicly available.

When an article is published we post the peer reviewers' comments and the authors' responses online. We also post the versions of the paper that were used during peer review. These are the versions that the peer review comments apply to.

The versions of the paper that follow are the versions that were submitted during the peer review process. They are not the versions of record or the final published versions. They should not be cited or distributed as the published version of this manuscript.

BMJ Open is an open access journal and the full, final, typeset and author-corrected version of record of the manuscript is available on our site with no access controls, subscription charges or pay-per-view fees (http://bmjopen.bmj.com).

If you have any questions on BMJ Open's open peer review process please email info.bmjopen@bmj.com

BMJ Open

Impact of the Tier system on SARS-CoV-2 transmission in the UK between the first and second national lockdowns

Journal:	*BMJ Open*
Manuscript ID	bmjopen-2021-050346
Article Type:	Original research
Date Submitted by the Author:	17-Feb-2021

Complete List of Authors:	Laydon, Daniel; Imperial College London, Department of Infectious Disease Epidemiology, MRC Centre for Global Infectious Disease Analysis, Jameel-Institute for Disease and Emergency Analytics<br>Mishra, Swapnil ; Imperial College London, Department of Infectious Disease Epidemiology, MRC Centre for Global Infectious Disease Analysis, Jameel-Institute for Disease and Emergency Analytics<br>Hinsley, Wes; Imperial College London, Department of Infectious Disease Epidemiology, MRC Centre for Global Infectious Disease Analysis, Jameel-Institute for Disease and Emergency Analytics<br>Samartsidis, Pantelis; University of Cambridge, MRC Biostatistics Unit, Cambridge Institute of Public Health<br>Flaxman, Seth; Imperial College London, Department of Mathematics and Data Science Institute<br>Gandy, Axel; Imperial College London, Department of Mathematics<br>Ferguson, Neil; Imperial College London, Department of Infectious Disease Epidemiology, MRC Centre for Global Infectious Disease Analysis, Jameel-Institute for Disease and Emergency Analytics<br>Bhatt, Samir; Imperial College London, Department of Infectious Disease Epidemiology, MRC Centre for Global Infectious Disease Analysis, Jameel-Institute for Disease and Emergency Analytics

Keywords:	COVID-19, EPIDEMIOLOGY, INFECTIOUS DISEASES, Epidemiology < INFECTIOUS DISEASES

SCHOLARONE™
Manuscripts

I, the Submitting Author has the right to grant and does grant on behalf of all authors of the Work (as defined in the below author licence), an exclusive licence and/or a non-exclusive licence for contributions from authors who are: i) UK Crown employees; ii) where BMJ has agreed a CC-BY licence shall apply, and/or iii) in accordance with the terms applicable for US Federal Government officers or employees acting as part of their official duties; on a worldwide, perpetual, irrevocable, royalty-free basis to BMJ Publishing Group Ltd ("BMJ") its licensees and where the relevant Journal is co-owned by BMJ to the co-owners of the Journal, to publish the Work in this journal and any other BMJ products and to exploit all rights, as set out in our licence.

The Submitting Author accepts and understands that any supply made under these terms is made by BMJ to the Submitting Author unless you are acting as an employee on behalf of your employer or a postgraduate student of an affiliated institution which is paying any applicable article publishing charge ("APC") for Open Access articles. Where the Submitting Author wishes to make the Work available on an Open Access basis (and intends to pay the relevant APC), the terms of reuse of such Open Access shall be governed by a Creative Commons licence – details of these licences and which Creative Commons licence will apply to this Work are set out in our licence referred to above.

Other than as permitted in any relevant BMJ Author's Self Archiving Policies, I confirm this Work has not been accepted for publication elsewhere, is not being considered for publication elsewhere and does not duplicate material already published. I confirm all authors consent to publication of this Work and authorise the granting of this licence.

For peer review only

Impact of the Tier system on SARS-CoV-2 transmission in the UK between the first and second national lockdowns

Daniel J Laydon[1,*], Swapnil Mishra[1], Wes R Hinsley[1], Pantelis Samartsidis[2], Seth Flaxman[3], Axel Gandy[3], Neil M Ferguson[1], Samir Bhatt[1]

[1] Department of Infectious Disease Epidemiology, MRC Centre for Global Infectious Disease Analysis, Jameel-Institute for Disease and Emergency Analytics, Imperial College London, School of Public Health, Norfolk Place, London, W2 1PG

[2] MRC Biostatistics Unit, Cambridge Institute of Public Health, Forvie Site, Robinson Way, Cambridge Biomedical Campus, CB2 0SR, United Kingdom

[3] Department of Mathematics, Imperial College London, South Kensington Campus, London, SW7 2AZ

[*] ***Corresponding Author: d.laydon@imperial.ac.uk;***

For peer review only

Abstract

Objective: Measure the effects of the Tier system on the COVID-19 pandemic in the UK between the first and second national lockdowns, before the emergence of the B.1.1.7 variant of concern.

Design: Modelling study combining estimates of the real-time reproduction number R_t (derived from UK case, death and serological survey data) with publicly available data on regional non-pharmaceutical interventions. We fit a Bayesian hierarchical model with latent factors using these quantities, to account for broader national trends in addition to subnational effects from Tiers.

Setting: The UK at Lower Tier Local Authority (LTLA) level.

For peer review only - http://bmjopen.bmj.com/site/about/guidelines.xhtml

Primary and secondary outcome measures: Reduction in real-time reproduction number R_t.

Results: Nationally, transmission increased between July and late September, regional differences notwithstanding. Immediately prior to the introduction of the tier system, R_t averaged 1.3 (0.9 – 1.6) across LTLAs, but declined to an average of 1.1 (0.86 – 1.42) two weeks later. Decline in transmission was not solely attributable to Tiers. Tier 1 had negligible effects. Tiers 2 and 3 respectively reduced transmission by 6% (5%-7%) and 23% (21%-25%). 93% of LTLAs would have begun to suppress their epidemics if every LTLA had gone into Tier 3 by the second national lockdown, whereas only 29% did so in reality.

Conclusions: The relatively small effect sizes found in this analysis demonstrate that interventions at least as stringent as Tier 3 are required to suppress transmission, especially considering more transmissible variants, at least until effective vaccination is widespread or much greater population immunity has amassed.

Strengths and limitations of this study

- First study to measure effects of UK Tier system for SARS-CoV-2 control at national and regional level.
- Model makes minimal assumptions and is primarily data driven.
- Insufficient statistical power to estimate effects of individual interventions that comprise Tiers, or their interaction.
- Estimates show that Tiers 1 and 2 are insufficient to suppress transmission, at least until widespread population immunity has amassed. Emergence of more transmissible variants of concern unfortunately supports this conclusion.

For peer review only - http://bmjopen.bmj.com/site/about/guidelines.xhtml

Introduction

There is substantial evidence supporting the effectiveness of government mandated social distancing (so-called “lockdown”) measures [1-5] in suppressing SARS-CoV-2 transmission. The massive economic and social cost of these measures in the United Kingdom motivated the less socially disruptive Tier system, introduced on 14th October 2020 [6]. Its aim was to provide a consistent set of COVID-19 control measures with geographic flexibility. Tiers consist of multiple non-pharmaceutical interventions (NPIs), and were determined by Lower Tier Local Authorities (LTLAs) in response to their local transmission intensity. Tiers were inconsistently defined across LTLAs, e.g. some LTLAs left gyms and fitness centres open under in Tier 1, while most did not. There is currently little evidence of the effectiveness or otherwise of the UK Tier system, which ran until the second national lockdown on 5th November 2020, before the emergence of the more transmissible variant of concern B.1.1.7.

Estimating the effect of the Tiers on underlying transmission is challenging, due to lags between their implementation and any change in cases, deaths, and the results of serological surveys. Therefore, estimating effect sizes by using only raw data can produce spurious results. Semi-mechanistic modelling that combines a transmission model with statistical modelling of transmission provides an alternative. There are two common approaches, estimating effect sizes directly within a semi-mechanistic transmission model [1], or estimating effect sizes in two stages [4]. by first nonparametrically estimating R_t and then performing a more classical regression analysis. Both approaches have their strengths and weaknesses from a statistical viewpoint. From a computational perspective, however, full Bayesian modelling of a

semi-mechanistic model across more than 300 LTLAs is not tractable. In this work, we therefore adopt a two-stage approach. Leveraging our existing LTLA transmission intensity estimation platform [7], we first estimate R_t (the rate of transmission, i.e. the number of secondary infections per infection at time t) using data on cases, deaths and serology. We model R_t as a weekly random process, making no assumptions on the effect or timing of interventions, and incorporate a wide range of data synthesis mechanisms. We further collate publicly available data on NPIs and Tiers at LTLA level. We combine these two sources of information to fit a Bayesian hierarchical model and estimate the effects of the UK Tier system between the first and second national lockdowns.

Methods

Collation of intervention data by LTLA

We compiled a list of the interventions implemented by each LTLA over time between 1st July 2020 and 5th November 2020. Data was obtained by monitoring the following government sources:

- https://www.gov.uk/government/collections/local-restrictions-areas-with-an-outbreak-of-coronavirus-covid-19
- https://www.gov.uk/guidance/full-list-of-local-covid-alert-levels-by-area,

and amending the interventions and Tiers that LTLAs were subject to accordingly.

For peer review only - http://bmjopen.bmj.com/site/about/guidelines.xhtml

Back-dating Tier classification

Since the Tier classification came into place on 14th October 2020, a strict evaluation of the effects of Tiers would limit our analysis to only 21 days until the second national lockdown on 5th November. However, the interventions that comprise a given Tier were in place earlier. We therefore retrospectively assign a Tier rating to LTLAs from 1st July 2020, when restrictions of the first national lockdown were lifted for the majority of the UK. The challenge is that the exact interventions that were implemented under Tiers 1, 2 and 3 varied firstly between LTLAs, and secondly over time. We determine the interventions that comprise a given Tier (and thus which Tier a given LTLA would have been in before the creation of the Tier system) using the number of LTLAs that implemented a given intervention on the day Tiers 1-3 were applied.

Real-time reproduction number estimates

Full details of the R_t estimation can be found in [7]. Briefly however, the method estimates transmission by calculating backwards from observed deaths (day of death), cases and serological survey data while simultaneously allowing for the time lag between infection and death. Infection fatality ratios and infection ascertainment rates are also calibrated nationally. The method accounts for noise in the data, noise in the stochastic process, lags in reporting, and day of the week variation.

Hierarchical Model

With R_t estimates for weekly time points $t = 1, \ldots, \tau$ (τ = 19 weeks between July 1st 2020 and November 4th 2020) and for each individual LTLA indexed by $m = 1, \ldots M$, ($M = 310$ individual local authorities), we use a regression model to estimate the effect

For peer review only - http://bmjopen.bmj.com/site/about/guidelines.xhtml

size of the introduction of Tiers. Specifically, for each LTLA m, we have a response variable $y_m \in \mathbb{R}^{\tau}$ of R_t values, and a binary design matrix $x_m \in 1\mathbb{R}^{\tau \times 3}$, where each column corresponds to the back dated Tier 1, 2 or 3, and the indicator function is a binary toggle for whether an LTLA is in a given Tier on a given date. Defining Tiers cumulatively (e.g. if an LTLA is in Tier 2 then it is also in Tier 1), makes little difference to our results. We propose a latent factor model that estimates the effect of Tiers, but that also accounts for confounding from other factors. We name these other factors secular trends, and they represent changes in transmission due to individual or wider societal behavioural changes along with LTLA idiosyncrasies and demographics, that are not part of the formal Tier system.

The linear model specification is:

$$\begin{gathered} y_m \sim Normal(\mu_m, \sigma) \\ \mu_m = \exp\left(\lambda\alpha_m^T - \beta x_m^T\right) \\ \beta \sim Normal^+(0, \phi_1) \\ \alpha_m \sim Normal^+(0, \phi_2) \\ \lambda \sim Normal^+(0, \phi_3) \\ \phi_1, \phi_2, \phi_3 \sim Normal^+(0,2) \\ \sigma \sim Normal^+(0,2) \end{gathered} \quad (130)$$

In the absence of LTLA-mandated interventions, a given LTLA *m* has a trend $\lambda\alpha_m^T$ in its reproduction number over time R_t. The above model is multiplicative: tiers reduce the R_t trend $\lambda\alpha_m^T$ by a factor of $\exp(-\beta x_m^T)$, for $\beta \in \mathbb{R}^3$. The secular trends $\lambda\alpha_m^T$ can be factorised into two basis functions with internal dimension *b*, i.e. $\lambda \in \mathbb{R}^{\tau \times b}$ and $\alpha_m \in \mathbb{R}^b$. This factorisation enforces shared trends across all LTLAs. The internal dimension *b* can be interpreted as the number of factors or categories that each LTLA has (to varying extents), in addition to the Tiers, that may influence transmission intensity. *b* can also be interpreted as the number of ways in which regional R_t values can differ

For peer review only - http://bmjopen.bmj.com/site/about/guidelines.xhtml

from national values. Model flexibility and complexity increases with *b,* and we set *b = 2.* Note that the λ has the same values for all LTLAs, while each α_m basis differs by LTLA *m*. The first column in the basis function λ is an intercept, and the second are τ independent parameters. Normal shrinkage priors are used for λ to balance over and under fitting. Conceptually, the intercept component of the basis matrix λ accounts for different LTLA starting values in R_t, which could be due to different demographics, behaviour, or contact patterns.

The second basis α_m scales and moves the national secular trend for a given LTLA. The secular trend broadly corresponds to changes over time due to behaviour and other national policies that are independent from the Tiers (such as "the rule of six"). However, the absolute impact of the national trend can vary by LTLA. That is, the overall shape of trend is shared nationally, but its magnitude and starting value can differ across LTLAs. As well as being relatively parsimonious, this formulation captures the interdependence of R_t values between LTLAs, while not assuming any *a priori* relationship between LTLAs.

It is possible to allow for a larger internal dimension value b, but given that our R_t estimates and back-dated Tiers consist of only 22 weeks of data for each LTLA, increasing b risks overfitting. With greater internal dimension, the model is too flexible and so the data will struggle to separate the effects of the Tiers βx_m^T from the underlying trend in each LTLA's transmission $\lambda\alpha_m^T$. Such additional model complexity spuriously nullifies the effects of Tiers. Alternative bases for λ such as b-splines could be used, but we opted for a non-structured basis to limit *a priori* assumptions. Partial pooling of

For peer review only - http://bmjopen.bmj.com/site/about/guidelines.xhtml

β, whereby the effects of Tiers would differ by LTLA, was investigated but did not produce significant differences between LTLAs.

We conducted a sensitivity analysis on the priors of the hyperparameters φ_1, φ_2, φ_3, and σ of the hierarchical model (which respectively inform values for Tier coefficients, LTLA-specific scaling of the secular trend, the secular trend itself, and random noise around the resulting R_t values). In our main model these are given as positive normal distributions with standard deviations of 2. However, allowing the standard deviation to take values 0.5, 1, or 2.5 made negligible difference to our results. Further, we investigated heavy tailed Cauchy distributions. While this resulted in a posterior distribution with greater curvature, it made negligible difference to estimated Tier coefficients. Our results are therefore robust to changes in the prior, and we therefore opted for normal distributions with standard deviations of 2 for our main model, as this prior is wide and uninformative.

We fit to R_t estimates by LTLA over time, and not to cases and deaths. NPIs and Tiers ultimately affect transmission, which in turn affects cases and deaths only after a delay. Therefore, regressing on the time-varying reproduction number is preferable as such delays do not need to be incorporated into the model.

Fitting was performed using the Bayesian software platform Stan [8], via the R package "rstan" [9] and R version 3.6.3 [10], using 2000 iterations with a warm-up of 500 iterations across 10 chains. R-hat and other diagnostics indicated a fully converged model. All model code and data sets are available from either the authors, or alternatively at https://github.com/ImperialCollegeLondon/covid19model.

For peer review only - http://bmjopen.bmj.com/site/about/guidelines.xhtml

Results

Figure 1 shows R_t estimates the day before the Tier system was introduced and two weeks afterwards. On 13th October, R_t averaged 1.3 (0.9 – 1.6) across LTLAs, but by 28th October had declined to 1.1 (0.86 – 1.42).

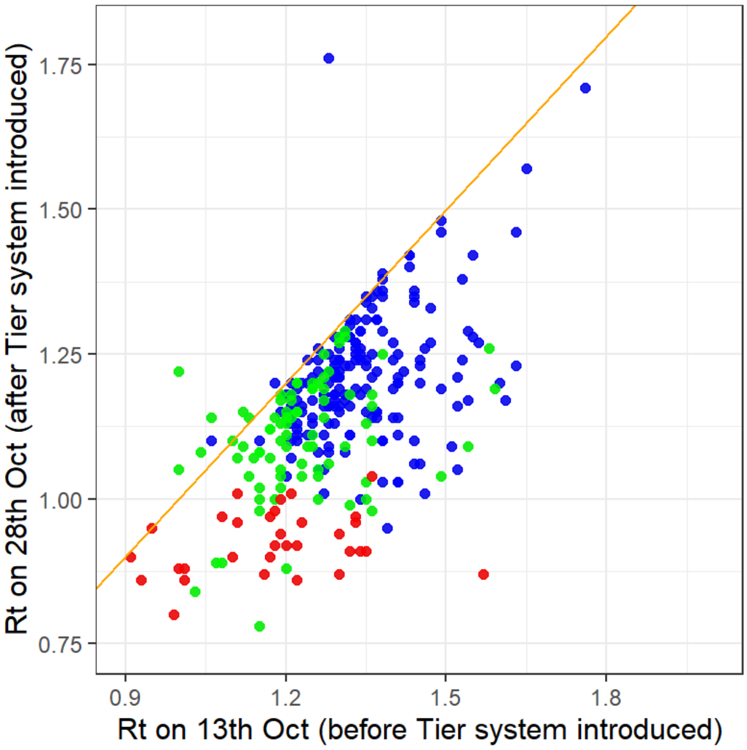

Figure 1: *R_t by LTLA one day before and two weeks after introduction of the Tier System. Blue points are Tier 1, green points are Tier 2 and red points are Tier 3. Line of equality shown in orange.*

Figure 2 shows the number of LTLAs that implemented a given intervention when nominally under Tiers 1, 2 and 3, at the earliest date they were introduced. Four interventions are implemented in all 310 LTLAs when under Tier 1: i) limiting indoor gatherings to at most six people; ii) limiting outdoor gatherings to at most six people; iii) curfew of 10pm for hospitality venues; iv) instruction to work from home where

For peer review only - http://bmjopen.bmj.com/site/about/guidelines.xhtml

possible. A further three interventions ("travel discouraged", "no indoor mixing", "overnight stays discouraged") are considered to be part of Tier 1 for 30 LTLAs. However, as this is minority of LTLAs, and as these interventions are always present under Tier 2, we include them in the definition of Tier 2. Tier 3 is the most consistently defined set of interventions, and adds the following two interventions to Tier 2: "residents cannot leave the local area" and "pubs and bars closed table service only". Closure of gyms is included in Tier 3 for six LTLAs, but this is insufficiently frequent to warrant inclusion in the backdating of Tier 3.

For peer review only

For peer review only - http://bmjopen.bmj.com/site/about/guidelines.xhtml

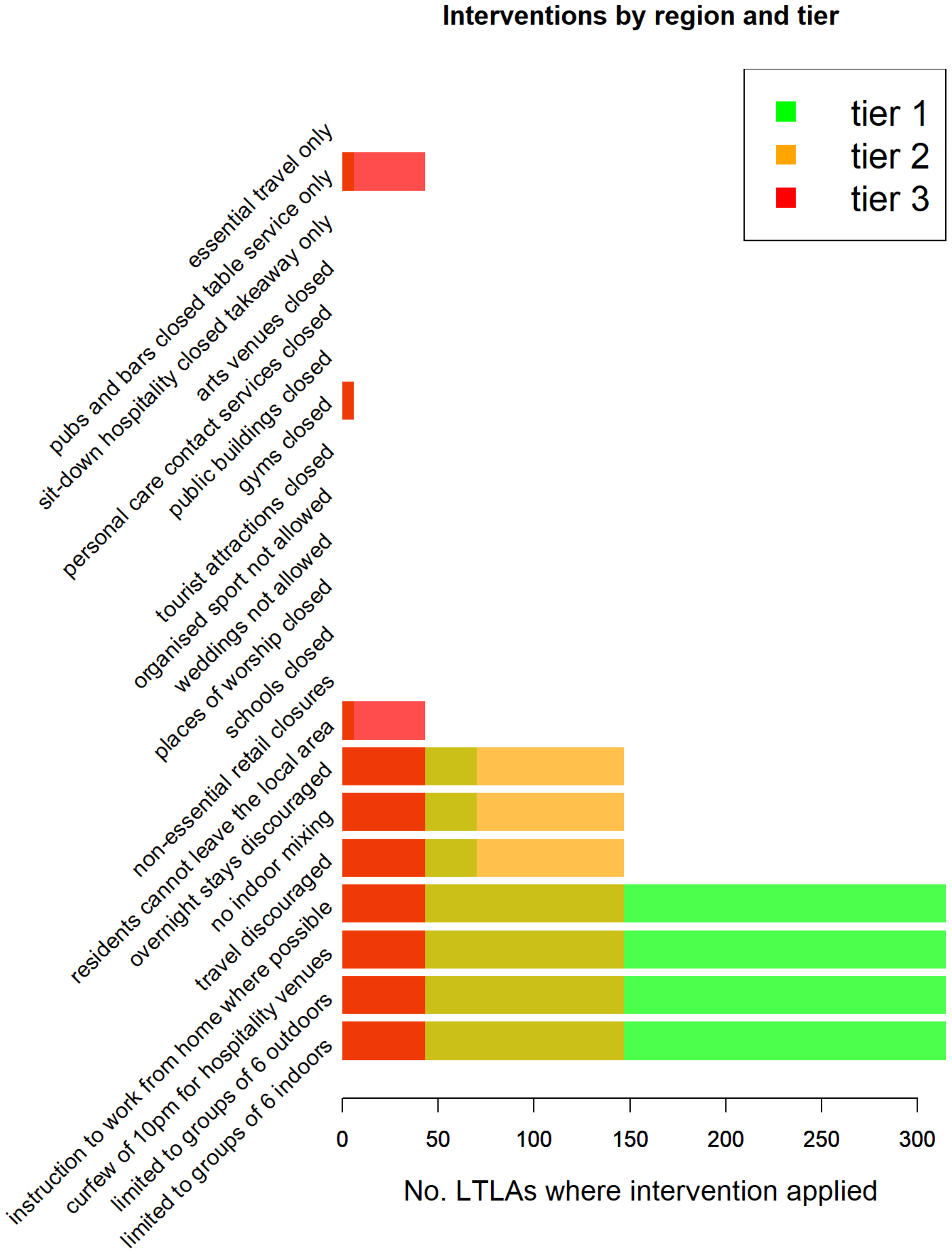

Figure 2: *Number of LTLAs applying interventions at introduction of Tier 1* ***(green)****, Tier 2* ***(yellow)****, or Tier 3* ***(red)****.*

Between July 1st and November 5th 2020, R_t took an average value of 1.13 (95% percentiles between 0.74 and 1.62) across all LTLAs. The latent factor secular trend accounts for much of the variation in R_t. The mean absolute error for our model is

For peer review only - http://bmjopen.bmj.com/site/about/guidelines.xhtml

8.7%, and without latent factors is 14%. The overall model fit (Figure 3, top left) suggests our model can reproduce trends in R_t relatively accurately (87% correlation). The secular trends (Figure 3, bottom right) suggest that transmission increased through to mid-September, when it began to reach a plateau. Transmission started to decline from late September, i.e. before the Tier system was introduced on October 14th. This implies that reduction in transmission is partly attributable to secular trends, independent of Tiers.

For peer review only

For peer review only - http://bmjopen.bmj.com/site/about/guidelines.xhtml

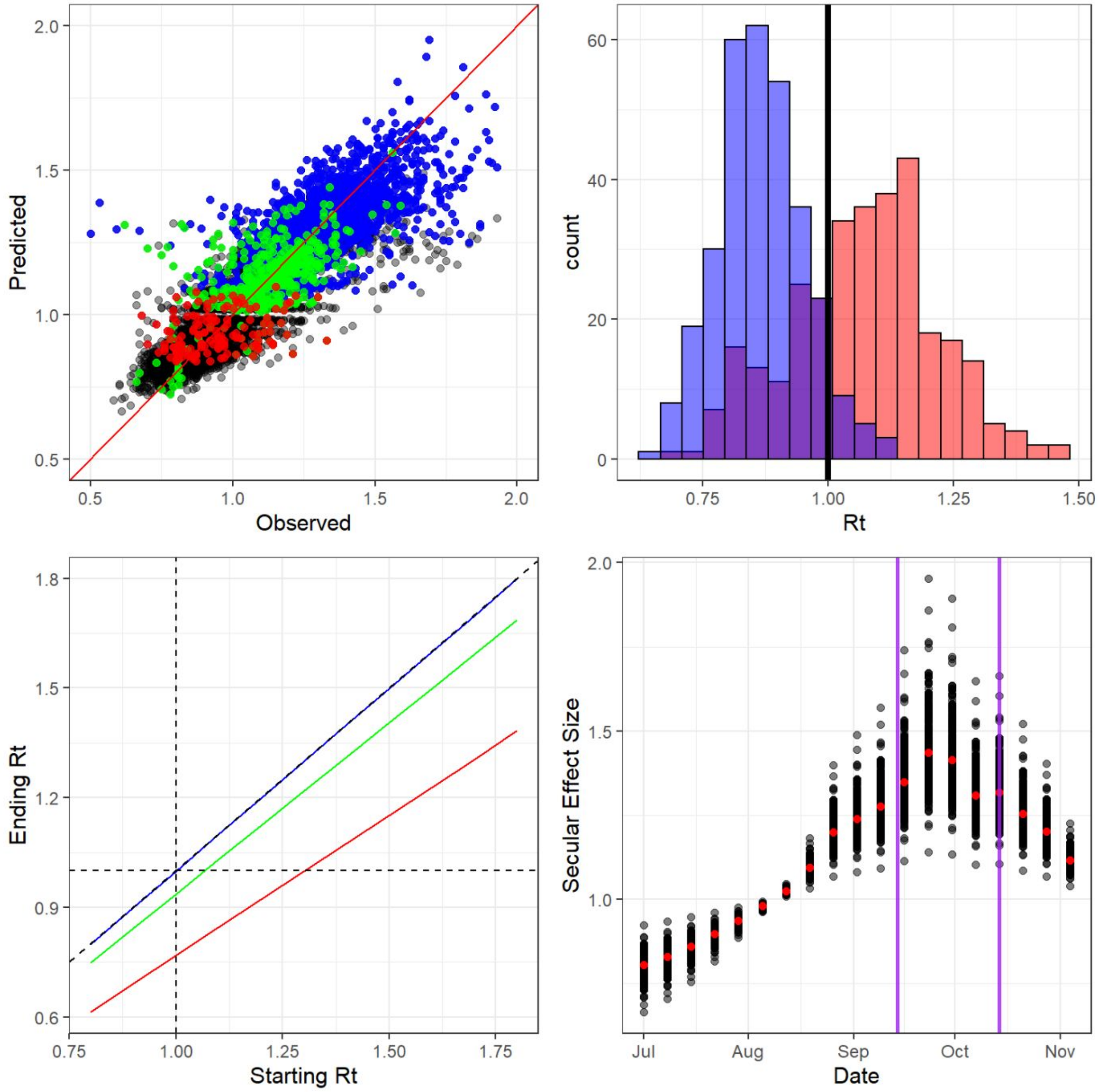

Figure 3: ***(Top Left)*** *Observed versus predicted* R_t. *Mean absolute error of 10% correlation of 63%. Blue points are Tier 1, green are Tier 2 and red are Tier 3. Black points are dates before the Tier system introduction.* ***(Top Right)*** *Histogram of* R_t *on 4th November 2020 for all LTLAs. Red bars are actual* R_t *values, blue bars are* R_t *if all LTLAS entered Tier 3.* ***(Bottom Left)*** *Black line is* $R_t = 1$ *the implied starting and ending* R_t *for a given Tier blue is Tier 1, green is Tier 2 and red is Tier 3 and the dotted lines are the 1 to 1 line and* $R_t = 1$. ***(Bottom Right)*** *Secular National trend from the latent factor models. Each dot for each week represents the mean effect size for a given LTLA and the red dots are the mean across all LTLA's. The first purple line corresponds to the rule of 6 being introduced and the second purple line corresponds to the formal introduction of the Tier system.*

For peer review only - http://bmjopen.bmj.com/site/about/guidelines.xhtml

Tier coefficients can be interpreted as a relative reduction in R_t. Therefore if R_t = 1, in the absence of Tiers, the reproduction number after entering a given Tier is given by the exponentiated coefficients in Table 1. That is, for any value of R, the reduction from Tier *i*, relative to the secular trend, is simply $R \times \exp(-\beta_i)$ for Tier coefficient β_i (Table 1). We detected no discernible effect from Tier 1 in addition to the secular trend, with β_1 = 0.001 (1.3 x 10^{-5} - 0.002), scaling R_t by 1. Tier 2 is slightly more effective and scales R_t by a factor of 0.94 (0.93 – 0.95). However, the effects of Tier 3 are significant, as it scales R_t by 0.77 (0.75 – 0.79).

Variable	Mean	2.5% CrI	97.5% CrI
Tier 1	1	1	1
Tier 2	0.94	0.93	0.95
Tier 3	0.77	0.75	0.79

Table 1: *Coefficient reductions from a given Tier. Note these have been exponentiated and can be interpreted by directly multiplying them with a given* R_t

It is important to stress that these estimates do not imply that the interventions comprising Tier 1 (e.g. working from home recommendations or limiting indoor gatherings to six people or less) would have zero effect, but rather that they have no additional effect beyond the secular trend. For example, Tier 1's prohibition of indoor or outdoor gatherings larger than six people was introduced on 14th October, after the more general "rule of six" that was already in place from 14th September.

For peer review only - http://bmjopen.bmj.com/site/about/guidelines.xhtml

We investigated what the effects on transmission would have been if all LTLAs had entered Tier 3. On November 4th 2020, only 28% of LTLAs were managing to suppress their epidemic with $R_t < 1$, with 95% of LTLAs having R_t values ranging from 0.79 to 1.35. Figure 3 (top right) shows the overall effect on R_t if all LTLAS had entered Tier 3 on November 4th, 2020. If all LTLAs had entered Tier 3 on November 5th, we estimate that 93% would have reduced R_t to less than 1, (95% credible interval of R_t values range between 0.66 and 1.03). In fact, only 29% of LTLAs managed to do so. These results suggest Tier 3 would have had a substantial effect on transmission, but would not conclusively reduce R_t below 1 in every LTLA. It should also be noted that if all LTLAs had been in Tier 3 on a different date, the effect would not have been the same due to secular trend changes.

Discussion

We estimated the effects of Tiers 1, 2 and 3 between the first and second national lockdowns in the UK. We estimated of the local time-varying reproduction number R_t using our established semi-mechanistic model. We combined these estimates with data detailing the timing of NPIs and Tiers at LTLA level to use as inputs in a Bayesian hierarchical model with a latent factor analysis.

Our approach aimed to account for broader secular trends at the national level in addition to the Tiers at LTLA level. The model reproduced R_t estimates reasonably well, and broadly captured their variation over time and variation between LTLAs. The national secular trend increased between early July and late September, notwithstanding regional differences and control measures applied. The national trend

For peer review only - http://bmjopen.bmj.com/site/about/guidelines.xhtml

began to plateau from mid-September (before the introduction of the Tier system) and declined shortly after, implying that transmission reduction was not solely attributable to Tiers. We find that Tier 1 has almost no effect on transmission beyond the secular national trend, and that Tier 2 yielded only minor reductions in transmission. However, Tier 3 was more effective, reducing transmission by an average of 23%. We estimated that, had Tier 3 been in effect throughout all LTLAs, suppression (R_t < 1) would have been achieved in 93% of LTLAs at the start of the second national lockdown, as opposed to the 29% of LTLAs that actually did manage to reduce R_t to below 1 at this time.

Our approach has a number of limitations. While the latent factors account for confounding to some degree, the coefficients we estimate are noncausal and therefore provide only associative effects. We do not consider the interaction of interventions, but merely their joint effect as mandated through the Tier system. In generating counterfactuals our model makes a "difference in differences" counterfactual assumption, which has previously been shown to have limitations [11] arising from the assumption of parallel trajectories. It is important to note that the effect sizes we model quantify the instantaneous and constant impact of the Tiers on R_t, whereas the effects of Tiers may vary over time, perhaps with a lag before they take effect or with a waning of efficacy. Our backdating of Tiers is imperfect: government announcement of a given Tier may have an additional effect beyond that of the particular NPIs within that Tier. However, it remains a reasonable approximation that enables the analysis of NPIs in finer detail than lockdown. It would be useful to measure the effects of the specific interventions that and comprise the Tiers, as this would enable more targeted

For peer review only - http://bmjopen.bmj.com/site/about/guidelines.xhtml

measures for COVID-19 control. However, the data is unfortunately insufficiently powered to make such inferences.

Our analysis focussed on the period between the first and second national lockdowns, before the emergence of the more-transmissible B.1.1.7 variant and before the rollout of vaccination. This focus avoids potential confounding factors of vaccination – it is impossible that we have falsely attributed the effects of vaccination to the impact of Tiers. While mass vaccination will enable less stringent interventions over time, the emergence of more transmissible variants will render Tiers 1 and 2 even less likely to be able to control SARS-CoV-2 transmission.

Non-pharmaceutical interventions will remain necessary to control SARS-CoV-2 transmission, particularly in light of newer more transmissible variants, and at least until an efficacious and effective vaccine becomes widely available or much greater population immunity has amassed. The relatively small effect sizes found in this analysis caution against expecting dramatic additional reductions in R_t from interventions less extreme than Tier 3.

Acknowledgements

We thank Joni Kirk and Pantelis Hadjipantelis of the Joint Biosecurity Centre, Department of Health & Social Care, and Shaun Seaman of the MRC Biostatistics Unit, Cambridge Institute of Public Health for helpful discussions.

For peer review only

Author Contributions

Conceptualization: DJL, SM, NMF, SB. **Data Curation**: DJL, SM, WRH, SF, AG, NMF, SB. **Formal Analysis**: DJL, SM, SF, AG, NMF, SB. **Funding Acquisition**: NMF, SB. **Investigation**: DJL, SM, NMF, SB. **Methodology**: DJL, SM, SF, PS, AG, NMF, SB. **Project administration**: NMF, SB. **Resources**: DJL, SM, WRH, SF, AG, NMF, SB. **Software**: DJL, SM, WRH, NMF, SB. **Supervision**: NMF, SB. **Validation**: DJL, SM, SF, AG, NMF, SB. **Visualisation**: DJL, NMF, SB. **Writing – original draft**: DJL, SB. **Writing – review & editing**: All authors reviewed the final draft.

Data Sharing Statement

All model code and data sets are available from either the authors, or alternatively at https://github.com/ImperialCollegeLondon/covid19model.

Funding Statement

DJL, NMF acknowledge funding from Vaccine Efficacy Evaluation for Priority Emerging Diseases (VEEPED) grant, (ref. NIHR: PR-OD-1017-20002) from the National Institute for Health Research. SB acknowledges the NIHR BRC Imperial College NHS Trust Infection and COVID themes, the Academy of Medical Sciences Springboard award (ref. SBF004\1080). DJL, SM, WRH, NMF and SB are supported by Centre funding (grant MR/R015600/1) from the UK Medical Research Council under a concordat with the UK Department for International Development, the NIHR Health Protection Research Unit in Modelling Methodology and Community Jameel. The funders of the study had no role in study design, data collection, data analysis, data interpretation, or writing of the report. The corresponding author had full access

For peer review only

For peer review only - http://bmjopen.bmj.com/site/about/guidelines.xhtml

to all the data in the study and had final responsibility for the decision to submit for publication.

Competing Interests

All authors have completed the Unified Competing Interest form (available on request from the corresponding author) and declare: no support from any organisation for the submitted work; no financial relationships with any organisations that might have an interest in the submitted work in the previous three years, no other relationships or activities that could appear to have influenced the submitted work.

For peer review only

For peer review only - http://bmjopen.bmj.com/site/about/guidelines.xhtml

References

1. Flaxman S, Mishra S, Gandy A, Unwin HJT, Mellan TA, Coupland H, et al. Estimating the effects of non-pharmaceutical interventions on COVID-19 in Europe. Nature. 2020;584(7820):257-61. doi: 10.1038/s41586-020-2405-7.

2. Salje H, Kiem CT, Lefrancq N, Courtejoie N, Bosetti P, Paireau J, et al. Estimating the burden of SARS-CoV-2 in France. 2020;369(6500):208-11.

3. Okell LC, Verity R, Watson OJ, Mishra S, Walker P, Whittaker C, et al. Have deaths from COVID-19 in Europe plateaued due to herd immunity? 2020;395(10241):e110-e1.

4. Haug N, Geyrhofer L, Londei A, Dervic E, Desvars-Larrive A, Loreto V, et al. Ranking the effectiveness of worldwide COVID-19 government interventions. Nature Human Behaviour. 2020;4(12):1303-12. doi: 10.1038/s41562-020-01009-0.

5. Fowler JH, Hill SJ, Obradovich N, Levin RJm. The Effect of Stay-at-Home Orders on COVID-19 Cases and Fatalities in the United States. 2020.

6. Iacobucci G. Covid-19: Three tier alert system takes effect across England. British Medical Journal Publishing Group; 2020.

7. Mishra S, Scott J, Zhu H, Ferguson NM, Bhatt S, Flaxman S, et al. A COVID-19 Model for Local Authorities of the United Kingdom. 2020:2020.11.24.20236661. doi: 10.1101/2020.11.24.20236661 %J medRxiv.

8. Carpenter B, Gelman A, Hoffman MD, Lee D, Goodrich B, Betancourt M, et al. Stan: a probabilistic programming language. 2017;76(1):1-32.

9. Team SD. RStan: the R interface to Stan (Version 2.17. 3). 2018.

10. Team RC. R: A Language and Environment for Statistical Computing. 467. 2020.

11. Samartsidis P, Seaman SR, Presanis AM, Hickman M, De Angelis DJSS. Assessing the causal effect of binary interventions from observational panel data with few treated units. 2019;34(3):486-503.

For peer review only - http://bmjopen.bmj.com/site/about/guidelines.xhtml

STROBE Statement—checklist of items that should be included in reports of observational studies

	Item No	Recommendation	Page No
**Title and abstract**	1	(*a*) Indicate the study's design with a commonly used term in the title or the abstract	1-2
		(*b*) Provide in the abstract an informative and balanced summary of what was done and what was found	1-2
**Introduction**			
Background/rationale	2	Explain the scientific background and rationale for the investigation being reported	3-4
Objectives	3	State specific objectives, including any prespecified hypotheses	3-4
**Methods**			
Study design	4	Present key elements of study design early in the paper	4-8
Setting	5	Describe the setting, locations, and relevant dates, including periods of recruitment, exposure, follow-up, and data collection	4-8
Participants	6	(*a*) *Cohort study*—Give the eligibility criteria, and the sources and methods of selection of participants. Describe methods of follow-up<br>*Case-control study*—Give the eligibility criteria, and the sources and methods of case ascertainment and control selection. Give the rationale for the choice of cases and controls<br>*Cross-sectional study*—Give the eligibility criteria, and the sources and methods of selection of participants	NA
		(*b*) *Cohort study*—For matched studies, give matching criteria and number of exposed and unexposed<br>*Case-control study*—For matched studies, give matching criteria and the number of controls per case	NA
Variables	7	Clearly define all outcomes, exposures, predictors, potential confounders, and effect modifiers. Give diagnostic criteria, if applicable	4-8
Data sources/ measurement	8*	For each variable of interest, give sources of data and details of methods of assessment (measurement). Describe comparability of assessment methods if there is more than one group	4-8
Bias	9	Describe any efforts to address potential sources of bias	4-8
Study size	10	Explain how the study size was arrived at	NA
Quantitative variables	11	Explain how quantitative variables were handled in the analyses. If applicable, describe which groupings were chosen and why	4-8
Statistical methods	12	(*a*) Describe all statistical methods, including those used to control for confounding	4-8
		(*b*) Describe any methods used to examine subgroups and interactions	NA
		(*c*) Explain how missing data were addressed	NA
		(*d*) *Cohort study*—If applicable, explain how loss to follow-up was addressed<br>*Case-control study*—If applicable, explain how matching of cases and controls was addressed<br>*Cross-sectional study*—If applicable, describe analytical methods taking account of sampling strategy	NA
		(*e*) Describe any sensitivity analyses	6,7

Continued on next page

For peer review only - http://bmjopen.bmj.com/site/about/guidelines.xhtml

**Results**			
Participants	13*	(a) Report numbers of individuals at each stage of study—eg numbers potentially eligible, examined for eligibility, confirmed eligible, included in the study, completing follow-up, and analysed	NA
		(b) Give reasons for non-participation at each stage	NA
		(c) Consider use of a flow diagram	NA
Descriptive data	14*	(a) Give characteristics of study participants (eg demographic, clinical, social) and information on exposures and potential confounders	NA
		(b) Indicate number of participants with missing data for each variable of interest	NA
		(c) *Cohort study*—Summarise follow-up time (eg, average and total amount)	NA
Outcome data	15*	*Cohort study*—Report numbers of outcome events or summary measures over time	NA
		Case-control study—Report numbers in each exposure category, or summary measures of exposure	NA
		Cross-sectional study—Report numbers of outcome events or summary measures	NA
Main results	16	(*a*) Give unadjusted estimates and, if applicable, confounder-adjusted estimates and their precision (eg, 95% confidence interval). Make clear which confounders were adjusted for and why they were included	8-14
		(*b*) Report category boundaries when continuous variables were categorized	NA
		(*c*) If relevant, consider translating estimates of relative risk into absolute risk for a meaningful time period	NA
Other analyses	17	Report other analyses done—eg analyses of subgroups and interactions, and sensitivity analyses	NA
**Discussion**			
Key results	18	Summarise key results with reference to study objectives	14
Limitations	19	Discuss limitations of the study, taking into account sources of potential bias or imprecision. Discuss both direction and magnitude of any potential bias	15-16
Interpretation	20	Give a cautious overall interpretation of results considering objectives, limitations, multiplicity of analyses, results from similar studies, and other relevant evidence	15-16
Generalisability	21	Discuss the generalisability (external validity) of the study results	16
**Other information**			
Funding	22	Give the source of funding and the role of the funders for the present study and, if applicable, for the original study on which the present article is based	17

*Give information separately for cases and controls in case-control studies and, if applicable, for exposed and unexposed groups in cohort and cross-sectional studies.

Note: An Explanation and Elaboration article discusses each checklist item and gives methodological background published examples of transparent reporting. The STROBE checklist is best used in conjunction with this article (freely available on the Web sites of PLoS Medicine at http://www.plosmedicine.org/, Annals of Internal Medicine at http://www.annals.org/, and Epidemiology at http://www.epidem.com/). Information on the STROBE Initiative is available at www.strobe-statement.org.

For peer review only - http://bmjopen.bmj.com/site/about/guidelines.xhtml

Modelling the impact of the Tier system on SARS-CoV-2 transmission in the UK between the first and second national lockdowns

Journal:	*BMJ Open*
Manuscript ID	bmjopen-2021-050346.R1
Article Type:	Original research
Date Submitted by the Author:	28-Mar-2021
Complete List of Authors:	Laydon, Daniel; Imperial College London, Department of Infectious Disease Epidemiology, MRC Centre for Global Infectious Disease Analysis, Jameel-Institute for Disease and Emergency Analytics<br>Mishra, Swapnil ; Imperial College London, Department of Infectious Disease Epidemiology, MRC Centre for Global Infectious Disease Analysis, Jameel-Institute for Disease and Emergency Analytics<br>Hinsley, Wes; Imperial College London, Department of Infectious Disease Epidemiology, MRC Centre for Global Infectious Disease Analysis, Jameel-Institute for Disease and Emergency Analytics<br>Samartsidis, Pantelis; University of Cambridge, MRC Biostatistics Unit, Cambridge Institute of Public Health<br>Flaxman, Seth; Imperial College London, Department of Mathematics and Data Science Institute<br>Gandy, Axel; Imperial College London, Department of Mathematics<br>Ferguson, Neil; Imperial College London, Department of Infectious Disease Epidemiology, MRC Centre for Global Infectious Disease Analysis, Jameel-Institute for Disease and Emergency Analytics<br>Bhatt, Samir; Imperial College London, Department of Infectious Disease Epidemiology, MRC Centre for Global Infectious Disease Analysis, Jameel-Institute for Disease and Emergency Analytics
**Primary Subject Heading**:	Epidemiology
Secondary Subject Heading:	Epidemiology, Infectious diseases, Public health
Keywords:	COVID-19, EPIDEMIOLOGY, INFECTIOUS DISEASES, Epidemiology < INFECTIOUS DISEASES

SCHOLARONE™
Manuscripts

I, the Submitting Author has the right to grant and does grant on behalf of all authors of the Work (as defined in the below author licence), an exclusive licence and/or a non-exclusive licence for contributions from authors who are: i) UK Crown employees; ii) where BMJ has agreed a CC-BY licence shall apply, and/or iii) in accordance with the terms applicable for US Federal Government officers or employees acting as part of their official duties; on a worldwide, perpetual, irrevocable, royalty-free basis to BMJ Publishing Group Ltd ("BMJ") its licensees and where the relevant Journal is co-owned by BMJ to the co-owners of the Journal, to publish the Work in this journal and any other BMJ products and to exploit all rights, as set out in our licence.

The Submitting Author accepts and understands that any supply made under these terms is made by BMJ to the Submitting Author unless you are acting as an employee on behalf of your employer or a postgraduate student of an affiliated institution which is paying any applicable article publishing charge ("APC") for Open Access articles. Where the Submitting Author wishes to make the Work available on an Open Access basis (and intends to pay the relevant APC), the terms of reuse of such Open Access shall be governed by a Creative Commons licence – details of these licences and which Creative Commons licence will apply to this Work are set out in our licence referred to above.

Other than as permitted in any relevant BMJ Author's Self Archiving Policies, I confirm this Work has not been accepted for publication elsewhere, is not being considered for publication elsewhere and does not duplicate material already published. I confirm all authors consent to publication of this Work and authorise the granting of this licence.

For peer review only

Modelling the impact of the Tier system on SARS-CoV-2 transmission in the UK between the first and second national lockdowns

Daniel J Laydon[1,*], Swapnil Mishra[1], Wes R Hinsley[1], Pantelis Samartsidis[2], Seth Flaxman[3], Axel Gandy[3], Neil M Ferguson[1], Samir Bhatt[1]

[1] Department of Infectious Disease Epidemiology, MRC Centre for Global Infectious Disease Analysis, Jameel-Institute for Disease and Emergency Analytics, Imperial College London, School of Public Health, Norfolk Place, London, W2 1PG

[2] MRC Biostatistics Unit, Cambridge Institute of Public Health, Forvie Site, Robinson Way, Cambridge Biomedical Campus, CB2 0SR, United Kingdom

[3] Department of Mathematics, Imperial College London, South Kensington Campus, London, SW7 2AZ

[*]***Corresponding Author:*** ***d.laydon@imperial.ac.uk;***

For peer review only

Abstract

Objective: Measure the effects of the Tier system on the COVID-19 pandemic in the UK between the first and second national lockdowns, before the emergence of the B.1.1.7 variant of concern.

Design: Modelling study combining estimates of the real-time reproduction number R_t (derived from UK case, death and serological survey data) with publicly available data on regional non-pharmaceutical interventions. We fit a Bayesian hierarchical model with latent factors using these quantities, to account for broader national trends in addition to subnational effects from Tiers.

Setting: The UK at Lower Tier Local Authority (LTLA) level. 310 LTLAs were included in the analysis

Primary and secondary outcome measures: Reduction in real-time reproduction number R_t.

Results: Nationally, transmission increased between July and late September, regional differences notwithstanding. Immediately prior to the introduction of the tier system, R_t averaged 1.3 (0.9 – 1.6) across LTLAs, but declined to an average of 1.1 (0.86 – 1.42) two weeks later. Decline in transmission was not solely attributable to Tiers. Tier 1 had negligible effects. Tiers 2 and 3 respectively reduced transmission by 6% (5%-7%) and 23% (21%-25%). 288 LTLAs (93%) would have begun to suppress their epidemics if every LTLA had gone into Tier 3 by the second national lockdown, whereas only 90 (29%) did so in reality.

Conclusions: The relatively small effect sizes found in this analysis demonstrate that interventions at least as stringent as Tier 3 are required to suppress transmission, especially considering more transmissible variants, at least until effective vaccination is widespread or much greater population immunity has amassed.

For peer review only

Strengths and limitations of this study

- First study to measure effects of UK Tier system for SARS-CoV-2 control at national and regional level.
- Model makes minimal assumptions and is primarily data driven.
- Insufficient statistical power to estimate effects of individual interventions that comprise Tiers, or their interaction.

For peer review only - http://bmjopen.bmj.com/site/about/guidelines.xhtml

Introduction

There is substantial evidence supporting the effectiveness of government mandated social distancing (so-called “lockdown”) measures [1-5] in suppressing SARS-CoV-2 transmission. The massive economic and social cost of these measures in the United Kingdom motivated the less socially disruptive Tier system, introduced on 14th October 2020 [6]. Its aim was to provide a consistent set of COVID-19 control measures with geographic flexibility. Tiers consist of multiple non-pharmaceutical interventions (NPIs), and were determined by Lower Tier Local Authorities (LTLAs) in response to their local transmission intensity. Tiers were inconsistently defined across LTLAs, e.g. some LTLAs left gyms and fitness centres open under in Tier 1, while most did not. There is currently little evidence of the effectiveness or otherwise of the UK Tier system, which ran until the second national lockdown on 5th November 2020, before the emergence of the more transmissible variant of concern B.1.1.7 and before Tier 4 was enacted.

Estimating the effect of the Tiers on underlying transmission is challenging, due to lags between their implementation and any change in cases, deaths, and the results of serological surveys. Therefore, estimating effect sizes by using only raw data can produce spurious results. Semi-mechanistic modelling that combines a transmission model with statistical modelling of transmission provides an alternative. There are two common approaches, estimating effect sizes directly within a semi-mechanistic transmission model [1], or estimating effect sizes in two stages [4]. by first nonparametrically estimating R_t and then performing a more classical regression analysis. Both approaches have their strengths and weaknesses from a statistical

For peer review only - http://bmjopen.bmj.com/site/about/guidelines.xhtml

viewpoint. From a computational perspective, however, full Bayesian modelling of a semi-mechanistic model across more than 300 LTLAs is not tractable. In this work, we therefore adopt a two-stage approach. Leveraging our existing LTLA transmission intensity estimation platform [7], we first estimate R_t (the rate of transmission, i.e. the number of secondary infections per infection at time *t*) using data on cases, deaths and serology. We model R_t as a weekly random process, making no assumptions on the effect or timing of interventions, and incorporate a wide range of data synthesis mechanisms. We further collate publicly available data on NPIs and Tiers at LTLA level. We combine these two sources of information to fit a Bayesian hierarchical model and estimate the effects of the UK Tier system between the first and second national lockdowns.

Methods

Collation of intervention data by LTLA

We compiled a list of the interventions implemented by each LTLA over time between 1st July 2020 and 5th November 2020. Data was obtained by monitoring the following government sources:

- https://www.gov.uk/government/collections/local-restrictions-areas-with-an-outbreak-of-coronavirus-covid-19
- https://www.gov.uk/guidance/full-list-of-local-covid-alert-levels-by-area,

and amending the interventions and Tiers that LTLAs were subject to accordingly.

For peer review only - http://bmjopen.bmj.com/site/about/guidelines.xhtml

Back-dating Tier classification

Since the Tier classification came into place on 14th October 2020, a strict evaluation of the effects of Tiers would limit our analysis to only 21 days until the second national lockdown on 5th November. However, the interventions that comprise a given Tier were in place earlier. We therefore retrospectively assign a Tier rating to LTLAs from 1st July 2020, when restrictions of the first national lockdown were lifted for the majority of the UK. The challenge is that the exact interventions that were implemented under Tiers 1, 2 and 3 varied firstly between LTLAs, and secondly over time. We determine the interventions that comprise a given Tier (and thus which Tier a given LTLA would have been in before the creation of the Tier system) using the number of LTLAs that implemented a given intervention on the day Tiers 1-3 were applied. Our study period ends before the second national lockdown and the implementation of Tier 4.

Figure 1 shows the number of LTLAs that implemented a given intervention when nominally under Tiers 1, 2 and 3, at the earliest date they were introduced. Four interventions are implemented in all 310 LTLAs when under Tier 1: i) limiting indoor gatherings to at most six people; ii) limiting outdoor gatherings to at most six people; iii) curfew of 10pm for hospitality venues; iv) instruction to work from home where possible. A further three interventions ("travel discouraged", "no indoor mixing", "overnight stays discouraged") are considered to be part of Tier 1 for 30 LTLAs. However, as this is minority of LTLAs, and as these interventions are always present under Tier 2, we include them in the definition of Tier 2. Tier 3 is the most consistently defined set of interventions, and adds the following two interventions to Tier 2: "residents cannot leave the local area" and "pubs and bars closed table service only".

For peer review only - http://bmjopen.bmj.com/site/about/guidelines.xhtml

Closure of gyms is included in Tier 3 for six LTLAs, but this is insufficiently frequent to warrant inclusion in the backdating of Tier 3.

Real-time reproduction number estimates

Full details of the R_t estimation can be found in [7]. Briefly however, the method estimates transmission by calculating backwards from observed deaths (day of death), cases and serological survey data while simultaneously allowing for the time lag between infection and death. Infection fatality ratios and infection ascertainment rates are also calibrated nationally. The method accounts for noise in the data, noise in the stochastic process, lags in reporting, and day of the week variation.

Hierarchical model

With R_t estimates for weekly time points $t = 1, \ldots, \tau$ (τ = 19 weeks between July 1st 2020 and November 4th 2020) and for each individual LTLA indexed by $m = 1, \ldots M$, ($M = 310$ individual local authorities), we use a regression model to estimate the effect size of the introduction of Tiers. Specifically, for each LTLA m, we have a response variable $y_m \in \mathbb{R}^{\tau}$ of R_t values, and a binary design matrix $x_m \in 1\mathbb{R}^{\tau \times 3}$, where each column corresponds to the back dated Tier 1, 2 or 3, and the indicator function is a binary toggle for whether an LTLA is in a given Tier on a given date. Defining Tiers cumulatively (e.g. if an LTLA is in Tier 2 then it is also in Tier 1), makes little difference to our results. We propose a latent factor model that estimates the effect of Tiers, but that also accounts for confounding from other factors. We name these other factors secular trends, and they represent changes in transmission due to individual or wider

For peer review only

For peer review only - http://bmjopen.bmj.com/site/about/guidelines.xhtml

societal behavioural changes along with LTLA idiosyncrasies and demographics, that are not part of the formal Tier system.

The linear model specification is:

$$
\begin{gathered}
y_m \sim Normal(\mu_m, \sigma) \\
\mu_m = \exp\left(\lambda\alpha_m^T - \beta x_m^T\right) \\
\beta \sim Normal^+(0, \phi_1) \\
\alpha_m \sim Normal^+(0, \phi_2) \\
\lambda \sim Normal^+(0, \phi_3) \\
\phi_1, \phi_2, \phi_3 \sim Normal^+(0, 2) \\
\sigma \sim Normal^+(0, 2)
\end{gathered}
$$

In the absence of LTLA-mandated interventions, a given LTLA *m* has a trend $\lambda\alpha_m^T$ in its reproduction number over time R_t. The above model is multiplicative: tiers reduce the R_t trend $\lambda\alpha_m^T$ by a factor of $\exp(-\beta x_m^T)$, for $\beta \in \mathbb{R}^3$. The secular trends $\lambda\alpha_m^T$ can be factorised into two basis functions with internal dimension *b*, i.e. a national trend $\lambda \in \mathbb{R}^{\tau \times b}$ and a regional scaling $\alpha_m \in \mathbb{R}^b$. This factorisation enforces shared trends across all LTLAs. The internal dimension *b* can be interpreted as the number of factors or categories that each LTLA has (to varying extents), in addition to the Tiers, that may influence transmission intensity. *b* can also be interpreted as the number of ways in which regional R_t values can differ from national values. Model flexibility and complexity increases with *b,* and we set *b* = 2*.* Note that the national trend λ has the same values for all LTLAs, while each α_m basis differs by LTLA *m*. The first column in the basis function λ is an intercept, and the second are τ independent parameters. Normal shrinkage priors are used for λ to balance over and under fitting. Conceptually, the intercept component of the basis matrix λ accounts for different LTLA starting values in R_t, which could be due to different demographics, behaviour, or contact patterns.

For peer review only

For peer review only - http://bmjopen.bmj.com/site/about/guidelines.xhtml

The second basis α_m scales and moves the national secular trend λ for a given LTLA *m*. The secular trend broadly corresponds to changes over time due to behaviour and other national policies that are independent from the Tiers (such as "the rule of six"). However, the absolute impact of the national trend can vary by LTLA. That is, the overall shape of trend is shared nationally, but its magnitude and starting value can differ across LTLAs. As well as being relatively parsimonious, this formulation captures the interdependence of R_t values between LTLAs, while not assuming any *a priori* relationship between LTLAs.

It is possible to allow for a larger internal dimension value b, but given that our R_t estimates and back-dated Tiers consist of only 22 weeks of data for each LTLA, increasing b risks overfitting. With greater internal dimension, the model is too flexible and so the data will struggle to separate the effects of the Tiers βx_m^T from the underlying trend in each LTLA's transmission $\lambda \alpha_m^T$. A similar problem arises if the priors for the β parameters are not restricted to non-negative values. Such additional model complexity spuriously nullifies the effects of Tiers. Alternative bases for λ such as b-splines could be used, but we opted for a non-structured basis to limit *a priori* assumptions. Partial pooling of β, whereby the effects of Tiers would differ by LTLA, was investigated but did not produce significant differences between LTLAs.

We conducted a sensitivity analysis on the priors of the hyperparameters φ_1, φ_2, φ_3, and σ of the hierarchical model (which respectively inform values for Tier coefficients, LTLA-specific scaling of the secular trend, the secular trend itself, and random noise around the resulting R_t values). In our main model these are given as positive normal

For peer review only - http://bmjopen.bmj.com/site/about/guidelines.xhtml

distributions with standard deviations of 2. However, allowing the standard deviation to take values 0.5, 1, or 2.5 made negligible difference to our results. Further, we investigated heavy tailed Cauchy distributions. While this resulted in a posterior distribution with greater curvature, it made negligible difference to estimated Tier coefficients. Our results are therefore robust to changes in the prior, and we therefore opted for normal distributions with standard deviations of 2 for our main model, as this prior is wide and uninformative.

We fit to R_t estimates by LTLA over time, and not to cases and deaths. NPIs and Tiers ultimately affect transmission, which in turn affects cases and deaths only after a delay. Therefore, regressing on the time-varying reproduction number is preferable as such delays do not need to be incorporated into the model.

Fitting was performed using the Bayesian software platform Stan [8], via the R package "rstan" [9] and R version 3.6.3 [10], using 2000 iterations with a warm-up of 500 iterations across 10 chains. R-hat and other diagnostics indicated a fully converged model. All model code and data sets are available from either the authors, or alternatively at https://github.com/ImperialCollegeLondon/covid19model.

Results

Figure 2 shows R_t estimates the day before the Tier system was introduced and two weeks afterwards. On 13th October, R_t averaged 1.3 (0.9 – 1.6) across LTLAs, but by 28th October had declined to 1.1 (0.86 – 1.42).

For peer review only - http://bmjopen.bmj.com/site/about/guidelines.xhtml

Between July 1st and November 5th 2020, R_t took an average value of 1.13 (95% percentiles between 0.74 and 1.62) across all LTLAs. The latent factor secular trend accounts for much of the variation in R_t. The mean absolute error for our model is 8.7%, and without latent factors is 14%. The overall model fit (Figure 3, top left) suggests our model can reproduce trends in R_t relatively accurately (87% correlation). The secular trends (Figure 3, bottom right) suggest that transmission increased through to mid-September, when it began to reach a plateau. Transmission started to decline from late September, i.e. before the Tier system was introduced on October 14th. This implies that reduction in transmission is partly attributable to secular trends, independent of Tiers. Tier coefficients can be interpreted as a relative reduction in R_t. Therefore if R_t = 1, in the absence of Tiers, the reproduction number after entering a given Tier is given by the exponentiated coefficients in Table 1. That is, for any value of R, the reduction from Tier *i*, relative to the secular trend, is simply $R \times \exp(-\beta_i)$ for Tier coefficient β_i (Table 1). We detected no discernible effect from Tier 1 in addition to the secular trend, with β_1 = 0.001 (1.3 x 10^{-5} - 0.002), scaling R_t by 1. Tier 2 is slightly more effective and scales R_t by a factor of 0.94 (0.93 – 0.95). However, the effects of Tier 3 are significant, as it scales R_t by 0.77 (0.75 – 0.79).

It is important to stress that these estimates do not imply that the interventions comprising Tier 1 (e.g. working from home recommendations or limiting indoor gatherings to six people or less) would have zero effect, but rather that they have no additional effect beyond the secular trend. For example, Tier 1's prohibition of indoor or outdoor gatherings larger than six people was introduced on 14th October, after the more general "rule of six" that was already in place from 14th September.

For peer review only - http://bmjopen.bmj.com/site/about/guidelines.xhtml

We investigated what the effects on transmission would have been if all LTLAs had entered Tier 3. On November 4th 2020, only 28% of LTLAs were managing to suppress their epidemic with $R_t < 1$, with 95% of LTLAs having R_t values ranging from 0.79 to 1.35. Figure 3 (top right) shows the overall effect on R_t if all LTLAS had entered Tier 3 on November 4th, 2020. If all LTLAs had entered Tier 3 on November 5th, we estimate that 288 LTLAs (93%) would have reduced R_t to less than 1, (95% credible interval of R_t values range between 0.66 and 1.03). In fact, only 90 LTLAs (29%) managed to do so. These results suggest Tier 3 would have had a substantial effect on transmission, but would not conclusively reduce R_t below 1 in every LTLA. It should also be noted that if all LTLAs had been in Tier 3 on a different date, the effect would not have been the same due to secular trend changes.

Discussion

Principal findings

We estimated the effects of Tiers 1, 2 and 3 between the first and second national lockdowns in the UK. We estimated of the local time-varying reproduction number R_t using our established semi-mechanistic model. We combined these estimates with data detailing the timing of NPIs and Tiers at LTLA level to use as inputs in a Bayesian hierarchical model with a latent factor analysis.

Our approach aimed to account for broader secular trends at the national level in addition to the Tiers at LTLA level. The model reproduced R_t estimates reasonably

For peer review only - http://bmjopen.bmj.com/site/about/guidelines.xhtml

259 well, and broadly captured their variation over time and variation between LTLAs. The
260 national secular trend increased between early July and late September,
261 notwithstanding regional differences and control measures applied. The national trend
262 began to plateau from mid-September (before the introduction of the Tier system) and
263 declined shortly after, implying that transmission reduction was not solely attributable
264 to Tiers. We find that Tier 1 has almost no effect on transmission beyond the secular
265 national trend, and that Tier 2 yielded only minor reductions in transmission. However,
266 Tier 3 was more effective, reducing transmission by an average of 23%. We estimated
267 that, had Tier 3 been in effect throughout all LTLAs, the real-time reproduction number
268 R_t would have been reduced to below 1 in 93% of LTLAs (288 LTLAs) at the start of
269 the second national lockdown, as opposed to the 29% of LTLAs (90 LTLAs) that
270 actually did manage to reduce R_t to below 1 at this time.

271

272 Limitations

273 Our approach has a number of limitations. While the latent factors account for
274 confounding to some degree, the coefficients we estimate are noncausal and therefore
275 provide only associative effects. We do not consider the interaction of interventions,
276 but merely their joint effect as mandated through the Tier system. In generating
277 counterfactuals our model makes a "difference in differences" counterfactual
278 assumption, which has previously been shown to have limitations [11] arising from the
279 assumption of parallel trajectories. It is important to note that the effect sizes we model
280 quantify the instantaneous and constant impact of the Tiers on R_t, whereas the effects
281 of Tiers may vary over time, perhaps with a lag before they take effect or with a waning
282 of efficacy.

283

For peer review only - http://bmjopen.bmj.com/site/about/guidelines.xhtml

Our backdating of Tiers is imperfect: government announcement of a given Tier may have an additional effect beyond that of the particular NPIs within that Tier. However, it remains a reasonable approximation that enables the analysis of NPIs in finer detail than lockdown. It would be useful to measure the effects of the specific interventions that and comprise the Tiers, as this would enable more targeted measures for COVID-19 control. However, the data is unfortunately insufficiently powered to make such inferences.

Further, our priors assume that Tiers will either reduce transmission or be ineffective, but do not allow Tiers to increase R_t. However, given that reduced human contact must reduce transmission, this is a reasonable assumption. We also do not propagate uncertainty in the reproduction number in the hierarchical model, as the high number of LTLAs render this computationally intractable. The development of frameworks that can propagate uncertainty reliably are important priorities for future work.

Conclusions

Our analysis focussed on the period between the first and second national lockdowns, before the emergence of the more-transmissible B.1.1.7 variant and before the rollout of vaccination. This focus avoids potential confounding factors of vaccination – it is impossible that we have falsely attributed the effects of vaccination to the impact of Tiers. While mass vaccination will enable less stringent interventions over time, the emergence of more transmissible variants will render Tiers 1 and 2 even less likely to be able to control SARS-CoV-2 transmission.

For peer review only - http://bmjopen.bmj.com/site/about/guidelines.xhtml

Non-pharmaceutical interventions will remain necessary to control SARS-CoV-2 transmission, particularly in light of newer more transmissible variants, and at least until an efficacious and effective vaccine becomes widely available or much greater population immunity has amassed. The relatively small effect sizes found in this analysis caution against expecting dramatic additional reductions in R_t from interventions less extreme than Tier 3.

Acknowledgements

We thank Joni Kirk and Pantelis Hadjipantelis of the Joint Biosecurity Centre, Department of Health & Social Care, and Shaun Seaman of the MRC Biostatistics Unit, Cambridge Institute of Public Health for helpful discussions.

Ethics statement

No ethical approval was necessary for this study because we use publicly available data.

Patient and public involvement

Patients and the public were not involved in the study design or analysis of this work.

Author contributions

Conceptualization: DJL, SM, NMF, SB. **Data Curation**: DJL, SM, WRH, SF, AG, NMF, SB. **Formal Analysis**: DJL, SM, SF, AG, NMF, SB. **Funding Acquisition**: NMF, SB. **Investigation**: DJL, SM, NMF, SB. **Methodology**: DJL, SM, SF, PS, AG, NMF,

For peer review only - http://bmjopen.bmj.com/site/about/guidelines.xhtml

For peer review only

SB. **Project administration**: NMF, SB. **Resources**: DJL, SM, WRH, SF, AG, NMF, SB. **Software**: DJL, SM, WRH, NMF, SB. **Supervision**: NMF, SB. **Validation**: DJL, SM, SF, AG, NMF, SB. **Visualisation**: DJL, NMF, SB. **Writing – original draft**: DJL, SB. **Writing – review & editing**: All authors reviewed the final draft.

Data sharing statement

All model code and data sets are available from either the authors, or alternatively at https://github.com/ImperialCollegeLondon/covid19model.

Funding statement

DJL, NMF acknowledge funding from Vaccine Efficacy Evaluation for Priority Emerging Diseases (VEEPED) grant, (ref. NIHR: PR-OD-1017-20002) from the National Institute for Health Research. SB acknowledges the NIHR BRC Imperial College NHS Trust Infection and COVID themes, the Academy of Medical Sciences Springboard award (ref. SBF004\1080). DJL, SM, WRH, NMF and SB are supported by Centre funding (grant MR/R015600/1) from the UK Medical Research Council under a concordat with the UK Department for International Development, the NIHR Health Protection Research Unit in Modelling Methodology and Community Jameel. The funders of the study had no role in study design, data collection, data analysis, data interpretation, or writing of the report. The corresponding author had full access to all the data in the study and had final responsibility for the decision to submit for publication.

For peer review only

Competing interests

All authors have completed the Unified Competing Interest form (available on request from the corresponding author) and declare: no support from any organisation for the submitted work; no financial relationships with any organisations that might have an interest in the submitted work in the previous three years, no other relationships or activities that could appear to have influenced the submitted work.

Tables

Variable	Mean	2.5% CrI	97.5% CrI
Tier 1	1	1	1
Tier 2	0.94	0.93	0.95
Tier 3	0.77	0.75	0.79

Table 1: Coefficient reductions from a given Tier. Note these have been exponentiated and can be interpreted by directly multiplying them with a given R_t.

For peer review only

For peer review only - http://bmjopen.bmj.com/site/about/guidelines.xhtml

Figure legends

Figure 1: Number of LTLAs applying interventions at introduction of: Tier 1 (**A**, green); Tier 2 (**B**, yellow); or Tier 3 (**C**, red).

Figure 2: R_t by LTLA one day before and two weeks after introduction of the Tier System. Blue points are Tier 1, green points are Tier 2 and red points are Tier 3. Line of equality shown in orange.

Figure 3: (Top Left) Observed versus predicted R_t. Mean absolute error of 10% correlation of 63%. Blue points are Tier 1, green are Tier 2 and red are Tier 3. Black points are dates before the Tier system introduction. **(Top Right)** Histogram of R_t on 4th November 2020 for all LTLAs. Red bars are actual R_t values, blue bars are R_t if all LTLAs entered Tier 3. **(Bottom Left)** Black line is $R_t = 1$ the implied starting and ending R_t for a given Tier blue is Tier 1, green is Tier 2 and red is Tier 3 and the dotted lines are the 1 to 1 line and $R_t = 1$. **(Bottom Right)** Secular National trend from the latent factor models. Each dot for each week represents the mean effect size for a given LTLA and the red dots are the mean across all LTLA's. The first purple line corresponds to the rule of 6 being introduced and the second purple line corresponds to the formal introduction of the Tier system.

For peer review only - http://bmjopen.bmj.com/site/about/guidelines.xhtml

References

1. Flaxman S, Mishra S, Gandy A, Unwin HJT, Mellan TA, Coupland H, et al. Estimating the effects of non-pharmaceutical interventions on COVID-19 in Europe. Nature. 2020;584(7820):257-61. doi: 10.1038/s41586-020-2405-7.

2. Salje H, Kiem CT, Lefrancq N, Courtejoie N, Bosetti P, Paireau J, et al. Estimating the burden of SARS-CoV-2 in France. 2020;369(6500):208-11.

3. Okell LC, Verity R, Watson OJ, Mishra S, Walker P, Whittaker C, et al. Have deaths from COVID-19 in Europe plateaued due to herd immunity? 2020;395(10241):e110-e1.

4. Haug N, Geyrhofer L, Londei A, Dervic E, Desvars-Larrive A, Loreto V, et al. Ranking the effectiveness of worldwide COVID-19 government interventions. Nature Human Behaviour. 2020;4(12):1303-12. doi: 10.1038/s41562-020-01009-0.

5. Fowler JH, Hill SJ, Obradovich N, Levin RJm. The Effect of Stay-at-Home Orders on COVID-19 Cases and Fatalities in the United States. 2020.

6. Iacobucci G. Covid-19: Three tier alert system takes effect across England. British Medical Journal Publishing Group; 2020.

7. Mishra S, Scott J, Zhu H, Ferguson NM, Bhatt S, Flaxman S, et al. A COVID-19 Model for Local Authorities of the United Kingdom. 2020:2020.11.24.20236661. doi: 10.1101/2020.11.24.20236661 %J medRxiv.

8. Carpenter B, Gelman A, Hoffman MD, Lee D, Goodrich B, Betancourt M, et al. Stan: a probabilistic programming language. 2017;76(1):1-32.

9. Team SD. RStan: the R interface to Stan (Version 2.17. 3). 2018.

10. Team RC. R: A Language and Environment for Statistical Computing. 467. 2020.

11. Samartsidis P, Seaman SR, Presanis AM, Hickman M, De Angelis DJSS. Assessing the causal effect of binary interventions from observational panel data with few treated units. 2019;34(3):486-503.

For peer review only

For peer review only - http://bmjopen.bmj.com/site/about/guidelines.xhtml

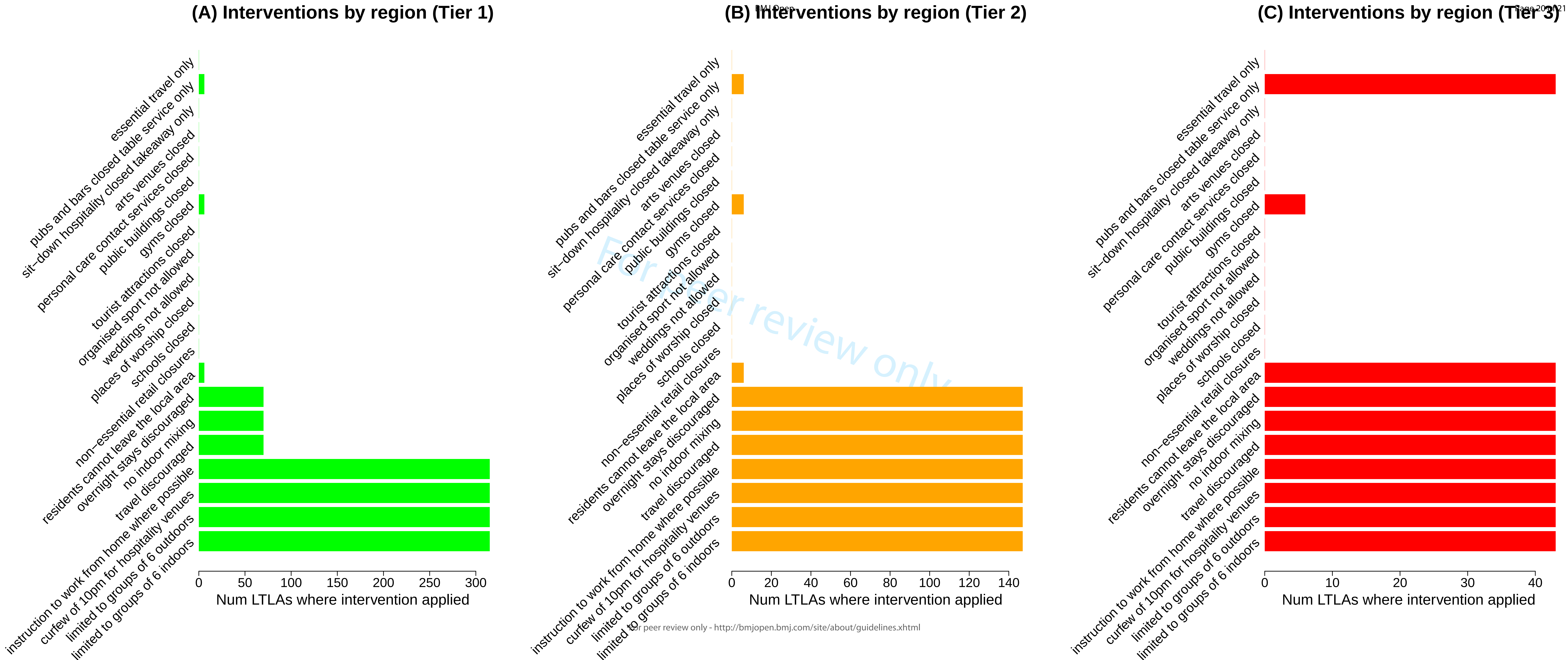

For peer review only

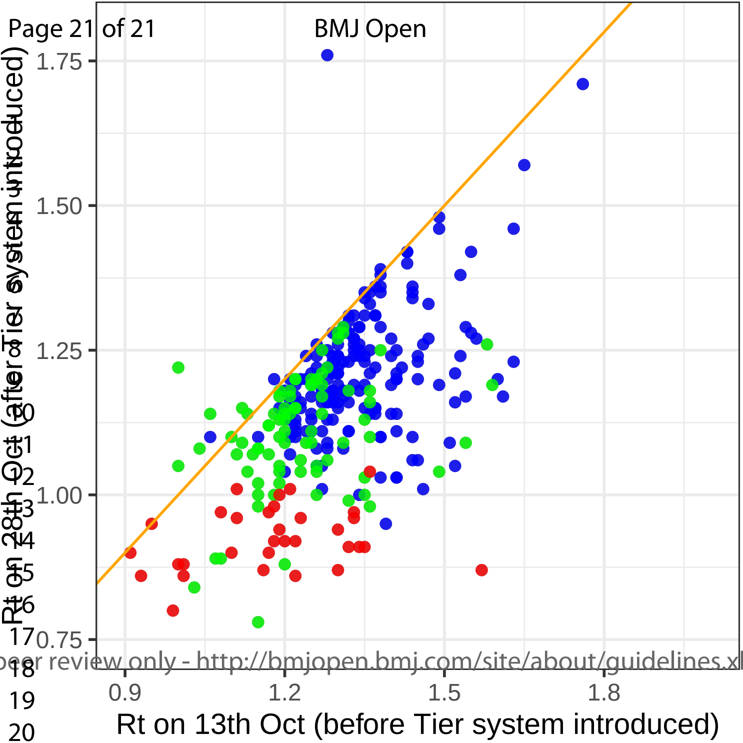

For peer review only - http://bmjopen.bmj.com/site/about/guidelines.xhtml

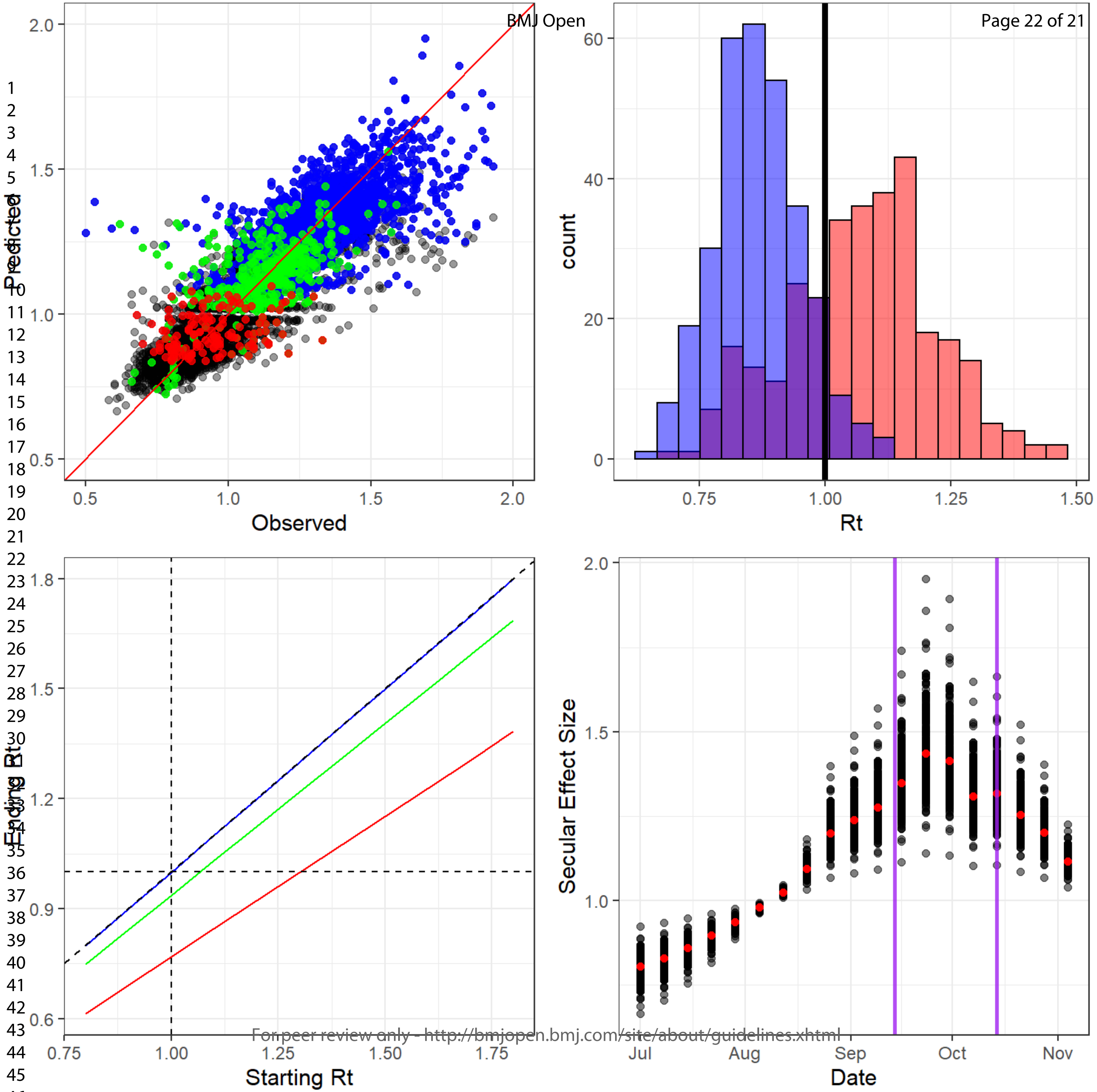
Predicted
Observed
count
Rt
Ending Rt
Starting Rt
Secular Effect Size
Date
Jul
Aug
Sep
Oct
Nov
